# Leukotriene receptor antagonist use and cognitive decline in normal cognition, mild cognitive impairment, and Alzheimer’s dementia

**DOI:** 10.1186/s13195-021-00892-7

**Published:** 2021-09-03

**Authors:** Lisa Y. Xiong, Michael Ouk, Che-Yuan Wu, Jennifer S. Rabin, Krista L. Lanctôt, Nathan Herrmann, Sandra E. Black, Jodi D. Edwards, Walter Swardfager

**Affiliations:** 1 Dr. Sandra Black Centre for Brain Resilience & Recovery, Hurvitz Brain Sciences Program, Sunnybrook Research Institute, Toronto, Canada; 2 Department of Pharmacology & Toxicology, University of Toronto, Room 4207, Medical Sciences Building, 1 King’s College Circle, Toronto, Ontario M5S 1A8 Canada; 3 Harquail Centre for Neuromodulation, Hurvitz Brain Sciences Program, Sunnybrook Research Institute, Toronto, Canada; 4 Department of Medicine (Neurology), Sunnybrook Health Sciences Centre, University of Toronto, Toronto, Canada; 5 Rehabilitation Sciences Institute, University of Toronto, Toronto, Canada; 6 Department of Psychiatry, Sunnybrook Health Sciences Centre, University of Toronto, Toronto, Canada; 7 KITE UHN Toronto Rehabilitation Institute, Toronto, Canada; 8 Canadian Partnership for Stroke Recovery, Toronto, Canada; 9 University of Ottawa Heart Institute, University of Ottawa, Ottawa, Canada; 10 School of Epidemiology and Public Health, University of Ottawa, Ottawa, Canada; 11 ICES, Ottawa, Canada

**Keywords:** Alzheimer’s disease, Mild cognitive impairment, Leukotriene receptor antagonists, Cognition, Language, Memory, Psychomotor processing, Clinical function

## Abstract

**Background:**

Leukotriene receptor antagonists (LTRAs) alleviate Alzheimer’s disease (AD) pathology and improve cognition in animal models; however, clinical evidence is limited. This study aimed to explore the associations between the use of LTRAs (montelukast or zafirlukast) and cognitive performance in people with normal cognition, mild cognitive impairment (MCI), or AD dementia. We hypothesized that LTRA use would be associated with better cognitive performance over time.

**Methods:**

This longitudinal observational study used data from the National Alzheimer’s Coordinating Center. Within groups of participants with normal cognition, MCI, or AD dementia, LTRA users were matched 1:3 to non-users using propensity score matching. Cognitive domains including immediate and delayed memory (Wechsler Memory Scale Revised-Logical Memory IA and IIA), psychomotor processing speed (Digit Symbol Substitution Test), and language (animal naming, vegetable naming, and Boston Naming Test) were compared between users and non-users in mixed-effects linear or Poisson regression models.

**Results:**

In AD dementia, LTRA use was associated with a slower decline in psychomotor processing speed, as measured by the Digit Symbol Substitution Test (*Β* = 1.466 [0.253, 2.678] symbols/year, *n* = 442), and language, as measured by animal naming (*Β* = 0.541 [0.215, 0.866] animals/year, *n* = 566), vegetable naming (*B* = 0.309 [0.056, 0.561] vegetables/year, *n* = 565), and the Boston Naming Test (*B* = 0.529 [0.005, 1.053] items/year, *n* = 561). Effect sizes were small but persisted after controlling for a 10% false discovery rate. LTRA use was not associated with changes in memory performance in AD, nor was it associated with changes in cognitive performance in people with normal cognition or MCI. In a post hoc analysis, LTRA use was associated with a slower decline in clinical progression in MCI (*B* = −0.200 [−0.380, −0.019] points/year, *n* = 800) and AD dementia (*B* = −0.321 [−0.597, −0.046] points/year, *n* = 604) as measured by CDR Sum of Boxes.

**Conclusions:**

The use of LTRAs was associated with preserved function in non-amnestic cognitive domains in AD dementia. The role of leukotrienes and their receptors in cognitive decline warrants further investigation and the leukotriene pathway may represent a target for AD treatment.

**Supplementary Information:**

The online version contains supplementary material available at 10.1186/s13195-021-00892-7.

## Background

Current treatments for Alzheimer’s disease (AD) are limited, and there is a need to explore potential mechanisms that might result in new treatment options. The leukotriene pathway is an inflammatory signaling pathway that has been implicated in multiple brain disorders including cerebral ischemia [1], dementia with Lewy bodies [2], and AD. In AD, proinflammatory responses mediated by leukotrienes have been suggested to modulate amyloid beta formation [3] and tau hyperphosphorylation [4]; therefore, repurposing leukotriene receptor antagonists (LTRAs) for AD is being explored [5–8].

LTRAs including montelukast and zafirlukast are indicated for asthma maintenance and allergic rhinitis. LTRAs exert their effects by inhibiting the binding of cysteinyl leukotrienes to their receptors, cysteinyl leukotriene receptor 1 (CysLTR1), and G-coupled protein receptor 17 (GPR17), thereby preventing proinflammatory signaling by the cysteinyl leukotrienes [9, 10]. It has been suggested that the pathophysiology of asthma and AD may be linked through activation of eicosanoid pathways which increase leukotriene production [11].

Preclinical animal studies suggest that administration of LTRAs may ameliorate AD-related pathology and associated cognitive decline [6–8]; however, human studies have been limited to three reports. One study on a large cohort of healthy people aged ≥ 60 years in Norway found that compared to use of inhaled corticosteroids, use of the LTRA montelukast was associated with a lower risk of dementia as indicated by the use of dementia medication (memantine, donepezil, rivastigmine, or galantamine). That study also found a lower risk of entering long-term care among people aged ≤ 75 [12]. A second study, also conducted in Norway, reported that compared to non-users, the use of montelukast was cross-sectionally associated with numerically but not significantly better performance in global cognition, psychomotor processing speed, and immediate free recall among a small sample (< 150) of older people [13]. Those results were among the first to suggest clinical benefits associated with the use of LTRAs, but the study may have been underpowered. In a case series of 17 dementia patients given montelukast, patients or their caregivers reported subjective improvements in memory and agitation [14]. Although these studies suggest possible benefits of LTRA use, there have been no longitudinal studies on LTRA use and objective cognitive performance, and no studies on LTRA use specifically among people with pre-existing mild cognitive impairment (MCI) or AD dementia.

The aim of this longitudinal study was to compare cognitive performance over time between users and non-users of an LTRA in a relatively larger sample of people with normal cognition, MCI, or AD dementia. We hypothesized that the use of an LTRA would be associated with a slower decline in cognitive performance over time.

## Methods

### Data source

The National Alzheimer’s Coordinating Center (NACC) has Alzheimer’s Disease Research Centers (ADRCs) located across the USA funded by the National Institute on Aging. The NACC maintains the Uniform Data Set (UDS) which is comprised of prospective, standardized, and longitudinal clinical data as previously described [15, 16]. Since its inception, there have been three versions of the UDS. Participants are recruited to the ADRCs through clinician referral, self-referral, or are recruited by community organizations. Data are collected approximately annually by trained clinicians and clinic personnel from participants and their co-participants (usually a close friend or family member) using a standardized evaluation via in-person office visits, home visits, and telephone calls.

### Participant selection

Data used in this analysis were collected between September 2005 and February 2021 from 42 ADRCs. Clinical diagnoses of cognitive status were made at each UDS visit by a single physician or consensus team. Participants were classified as cognitively normal if they had normal cognition and did not use any dementia-related medications (memantine, donepezil, rivastigmine, galantamine, or tacrine) at baseline. Participants were classified as MCI upon first diagnosis of MCI. Participants were classified as AD upon first diagnosis of all-cause dementia with AD being identified as a primary or contributing cause of cognitive impairment, as defined by NINCDS-ADRDA or NIA-AA criteria [17, 18]. Only participants who completed the medication form and had baseline data for the identified matching factors were included.

All-cause dementia was diagnosed using established criteria as previously described [17–19]. MCI was diagnosed using the Petersen’s criteria [20]. Normal cognition was defined as not exhibiting any dementia-related symptoms and having a global score of 0 from the CDR® Dementia Staging Instrument or neuropsychological testing within the normal range.

### Drug exposure

Medication use at each visit was identified by trained clinicians or ADRC staff using a structured medication inventory. Participants or co-participants were asked to bring or report all medications that were used within 2 weeks prior to each study visit. Participants who had used an LTRA (montelukast or zafirlukast) were included. LTRA users at baseline were compared to those who were never exposed to an LTRA.

The use of other classes of respiratory medications were also identified, including other medications for asthma maintenance, chronic obstructive pulmonary disease (COPD), allergies, or use of a rescue inhaler for asthma; these additional medications were included as covariates in the analysis. As measures of respiratory disease severity (e.g., spirometry) were not available in the database, these respiratory medications were used as a proxy for respiratory disease severity.

### Cognitive outcomes

The outcomes of interest were immediate and delayed logical memory, psychomotor processing speed, and language and were selected based on the findings from a previous study on montelukast and neurological function and the domains most affected by AD dementia [13, 21]. Immediate and delayed logical memory were assessed using the Wechsler Memory Scale Revised-Logical Memory Test IA (total number of story units recalled immediately) and IIA (total number of story units recalled after a 20-min delay) [22]. Psychomotor processing speed was measured by the Wechsler Adult Intelligence Scale-Revised (WAIS-R) Digit Symbol Substitution Test (number of correct symbols) [23]. Language was assessed using Animal naming (total number of animals named in 60 s), vegetable naming (total number of vegetables named in 60 s) [24], and the Boston Naming Test (total items named correctly) [25]. In all tests, a higher score indicates better performance.

Between versions 2 and 3 of the UDS, there was a change in the neuropsychological test battery, resulting in the replacement of some cognitive tests. New test scores in version 3 were converted to their equivalent scores on previous tests with the results from the Crosswalk Study [26] (i.e., Craft Immediate and Delayed Recall to Logical Memory Test IA and IIA and Multilingual Naming Test to Boston Naming Test).

### Statistical analysis

Analyses were conducted using R 4.0.5 [27] and figures were created using the ggplot2 package [28].

Propensity score matching (MatchIt package [29]) was performed to minimize confounding by indication and to create a group of non-users that was comparable to LTRA users in clinically important factors. Users were matched to non-users using the greedy nearest neighbor matching method in a 1:3 ratio. Matched groups were created for each of the normal cognition, MCI, and AD groups.

Propensity scores were calculated using age, sex, education, body mass index (BMI), smoking history (reported total years smoked > 0), use of other respiratory medications, apolipoprotein E (*APOE*) ε4 carrier status, use of dementia medications, CDR global score [30], and evidence of vascular contribution to dementia. Vascular contribution to dementia was defined by NINDS-AIREN criteria [31] or neurological evidence of cerebrovascular disease, cortical cognitive deficit (e.g., aphasia, apraxia, neglect), or subcortical ischemic vascular dementia as previously defined [32].

The associations between LTRA use and performance on immediate and delayed logical memory over time were assessed using mixed-effects Poisson regression models with random slopes (glmmTMB package [33]) as immediate and delayed logical memory are count variables following Poisson-like distributions. Effect sizes were reported as rate ratios (RRs), which indicate the fold-change in score over time. The associations between LTRA use and performance on the Digit Symbol Substitution Test, animal naming, vegetable naming, and Boston Naming Test were assessed using mixed-effects linear regression models with random slopes (lme4 package [34]). The mixed-effects models were able to handle missing follow-up data. Effect sizes were reported as unstandardized (Β) and standardized (β) regression coefficients. Analyses were performed separately for the normal cognition, MCI, and AD groups. To reduce potential residual confounding, the use of dementia medication at each visit and matching factors with standardized mean difference (SMD) ≥ 0.1 between users and non-users in the matched groups was included as covariates in the outcome models. Within each group, results were controlled for multiple comparisons using the Benjamini-Hochberg method [35] with a 10% false discovery rate (FDR).

Previous studies on the benefits of LTRAs in AD dementia have only included users of montelukast [12, 13]. To ensure that our results were comparable to those studies and not influenced by the inclusion of zafirlukast, a post hoc analysis was performed excluding the zafirlukast users by matching users of montelukast to non-users of an LTRA. To compare clinical progression in users and non-users of LTRAs, a separate post hoc analysis was performed in the MCI and AD groups with CDR Sum of Boxes (CDR-SOB) as an outcome.

## Results

### Participant characteristics

Of 43,746 participants, 11,976 participants met the criteria for inclusion into the normal cognition group, 7782 met the criteria for inclusion into the MCI group, and 8918 met the criteria for inclusion into the AD group. After conducting propensity score matching, the matched samples for normal cognition, MCI, and AD contained 1,400, 800, and 604 participants, respectively (participant selection flowchart shown in Figure S1). Baseline characteristics of each group after propensity score matching are presented in Table 1. The majority of LTRA users (99.8%) were using montelukast, and the remaining were using zafirlukast.

**Table 1:** Baseline participant characteristics by LTRA use in normal, MCI, and AD groups following propensity score matching

	Normal				MCI				AD			
	User (***n*** = 350)	Non-user (***n*** = 1050)	SMD	***p*** value	User (***n*** = 200)	Non-user (***n*** = 600)	SMD	***p*** value	User (***n*** = 151)	Non-user (***n*** = 453)	SMD	***p*** value
**Baseline demographics**												
Age (years)	70.30 (9.71)	70.37 (9.56)	0.007	0.904	73.75 (8.86)	73.94 (8.76)	0.021	0.795	75.35 (9.73)	75.24 (10.13)	0.011	0.907
Female	261 (74.6%)	793 (75.5%)	0.022	0.775	110 (55.0%)	321 (53.5%)	0.030	0.774	93 (61.6%)	265 (58.5%)	0.063	0.566
BMI (kg/m^2^)	29.41 (6.21)	29.70 (6.78)	0.045	0.475	27.82 (5.54)	27.92 (6.17)	0.018	0.831	27.03 (6.05)	26.93 (5.08)	0.018	0.842
Education (years)	15.77 (3.29)	15.81 (2.82)	0.013	0.822	15.23 (3.32)	15.15 (3.27)	0.024	0.770	13.87 (4.51)	14.03 (3.77)	0.038	0.670
Smoking history (total years smoked > 0)	158 (45.1%)	471 (44.9%)	0.006	0.975	86 (43.0%)	273 (45.5%)	0.050	0.594	60 (39.7%)	199 (43.9%)	0.085	0.420
**Dementia measures**												
CDR global score			0.019	0.838			0.065	0.697			0.119	0.659
0	316 (90.3%)	942 (89.7%)			25 (12.5%)	72 (12.0%)			0 (0.0%)	0 (0.0%)		
0.5	34 (9.7%)	108 (10.3%)			172 (86.0%)	523 (87.2%)			58 (38.4%)	200 (44.2%)		
1	0 (0.0%)	0 (0.0%)			3 (1.5%)	5 (0.8%)			61 (40.4%)	168 (37.1%)		
2	0 (0.0%)	0 (0.0%)			0 (0.0%)	0 (0.0%)			28 (18.5%)	73 (16.1%)		
3	0 (0.0%)	0 (0.0%)			0 (0.0%)	0 (0.0%)			4 (2.6%)	12 (2.6%)		
*APOE* ε4 carrier	116 (33.1%)	347 (33.0%)	0.002	1.000	84 (42.0%)	247 (41.2%)	0.017	0.901	81 (53.6%)	252 (55.6%)	0.040	0.741
Vascular dementia	0 (0.0%)	0 (0.0%)	< 0.001	-	7 (3.5%)	29 (4.8%)	0.067	0.555	6 (4.0%)	14 (3.1%)	0.048	0.793
AD medication use	0 (0.0%)	0 (0.0%)	< 0.001	-	52 (26.0%)	155 (25.8%)	0.004	> 0.999	100 (66.2%)	302 (66.7%)	0.009	> 0.999
**Other respiratory medication use**												
Allergy medications	152 (43.4%)	477 (45.4%)	0.040	0.556	82 (41.0%)	240 (40.0%)	0.020	0.868	51 (33.8%)	163 (36.0%)	0.046	0.694
COPD medications	27 (7.7%)	58 (5.5%)	0.088	0.175	27 (13.5%)	65 (10.8%)	0.082	0.370	17 (11.3%)	48 (10.6%)	0.021	0.940
Rescue inhaler for asthma	82 (23.4%)	178 (17.0%)	0.162	0.009	40 (20.0%)	129 (21.5%)	0.037	0.726	36 (23.8%)	92 (20.3%)	0.085	0.421
Maintenance inhaler for asthma	136 (38.9%)	350 (33.3%)	0.115	0.070	78 (39.0%)	211 (35.2%)	0.079	0.372	49 (32.5%)	140 (30.9%)	0.033	0.800

Continuous variables expressed as mean (standardized deviation); categorical variables expressed as counts (proportion)

Abbreviations: *AD* Alzheimer’s disease, *BMI* body mass index, *COPD* chronic obstructive pulmonary disease, *MCI* mild cognitive impairment, and *SMD* standardized mean difference

In the normal cognition sample, LTRA users (*n* = 350, median follow-up = 4.08 [1.79, 7.08] years, 86.3% with ≥ 2 observations) and non-users (*n* = 1050, median follow-up = 3.98 [1.59, 7.21] years, 85.9% with ≥ 2 observations) were balanced in clinical characteristics that were identified to be of importance except for small imbalances in the use of a rescue inhaler for asthma (SMD = 0.162) and use of a maintenance inhaler for asthma (SMD = 0.115), which were used by a greater proportion of LTRA users. These variables were included as covariates in the outcome model to account for possible residual confounding. The median follow-up time and proportion of participants with ≥ 2 observations did not differ significantly between users and non-users.

In the MCI sample, LTRA users (*n* = 200, median follow-up = 2.11 [0.96, 4.69] years, 77.5% with ≥ 2 observations) and non-users (*n* = 600, median follow-up = 2.46 [1.04, 4.91] years, 82.5% with ≥ 2 observations) were balanced in all identified clinical factors. The median follow-up time and proportion of participants with ≥ 2 observations did not differ significantly between users and non-users.

In the AD sample, LTRA users (*n* = 151, median follow-up = 1.92 [0.00, 3.84] years, 74.2% with ≥ 2 observations) and non-users (*n* = 453, median follow-up = 1.94 [0.00, 3.84] years, 72.0% with ≥ 2 observations) were balanced in all identified clinical factors except for a small imbalance in baseline CDR global score (SMD = 0.119), where a greater proportion of users had higher CDR global scores than non-users (i.e., had greater dementia severity). Baseline CDR global score was included as a covariate in the outcome model. The median follow-up time and proportion of participants with ≥ 2 observations did not differ significantly between users and non-users.

### Relationship between LTRA use and cognition in the matched sample with normal cognition

In the normal cognition sample, LTRA use was associated with a 0.8% faster rate of decline over time in immediate memory (RR = 0.992 [0.985, 0.998]) which remained statistically significant after controlling for FDR. No other differences in performance over time were observed between LTRA users and non-users (Table 2).

**Table 2:** Associations between LTRA use and cognitive test performance over time in the normal cognition group

	***n***		RR or Β [95% CI]	***β*** [95% CI]	***z*** or ***t***	***p*** value
	User	Non-user				
**Logical memory**						
Immediate memory	345	1040	RR = 0.992 [0.985, 0.998]	-	−2.55	.0106*
Delayed memory	345	1040	RR = 0.995 [0.987, 1.002]	-	−1.24	.2134
**Psychomotor processing speed**						
Digit Symbol Substitution Test	278	742	*B* = 0.158 [−0.099, 0.414]	0.012 [−0.007, 0.031]	1.21	.2284
**Language**						
Boston Naming Test	344	1041	*B* = −0.020 [−0.081, 0.041]	−0.007 [−0.026, 0.013]	−0.65	.5134
Animal naming	345	1043	*B* = 0.046 [−0.056, 0.148]	0.008 [−0.011, 0.026]	0.88	.3773
Vegetable naming	345	1039	*B* = −0.017 [−0.093, 0.062]	−0.004 −0.021, 0.014]	−0.40	.6912

Abbreviations: *RR* rate ratio, *B* unstandardized coefficient, *z* for logical memory, and *t* for all other tests

Significant at FDR 0.1

### Relationship between LTRA use and cognition in the matched sample with MCI

In the MCI sample, LTRA use was associated with a slower decline in psychomotor processing speed as measured by the Digit Symbol Substitution Test (*B* = 0.690 [0.035, 1.344] symbols/year); however, this finding did not remain significant after FDR correction. No other differences in performance over time were observed between LTRA users and non-users (Table 3).

**Table 3:** Associations between LTRA use and cognitive test performance over time in the MCI group

	***n***		RR or Β [95% CI]	β [95% CI]	***z*** or ***t***	***p*** value
	User	Non-user				
**Logical memory**						
Immediate memory	197	582	RR = 1.015 [0.991, 1.039]	-	1.24	.2150
Delayed memory	197	581	RR = 1.032 [0.993, 1.074]	-	1.59	.1120
**Psychomotor processing speed**						
Digit Symbol Substitution Test	150	431	*B* = 0.690 [0.035, 1.344]	0.055 [0.003, 0.107]	2.07	.0400
**Language**						
Boston Naming Test	197	584	*B* = 0.189 [−0.049, 0.427]	0.035 [−0.009, 0.079]	1.55	.1210
Animal naming	197	584	*B* = 0.189 [−0.043, 0.421]	0.034 [−0.008, 0.075]	1.59	.1120
Vegetable naming	197	581	*B* = 0.035 [−0.129, 0.200]	0.001 [−0.030, 0.047]	0.42	.6755

*RR* rate ratio, *B* unstandardized coefficient, *z* for logical memory, and *t* for all other tests

^*^Significant at FDR 0.1

### Relationship between LTRA use and cognition in the matched sample with AD dementia

**Fig. 1:**
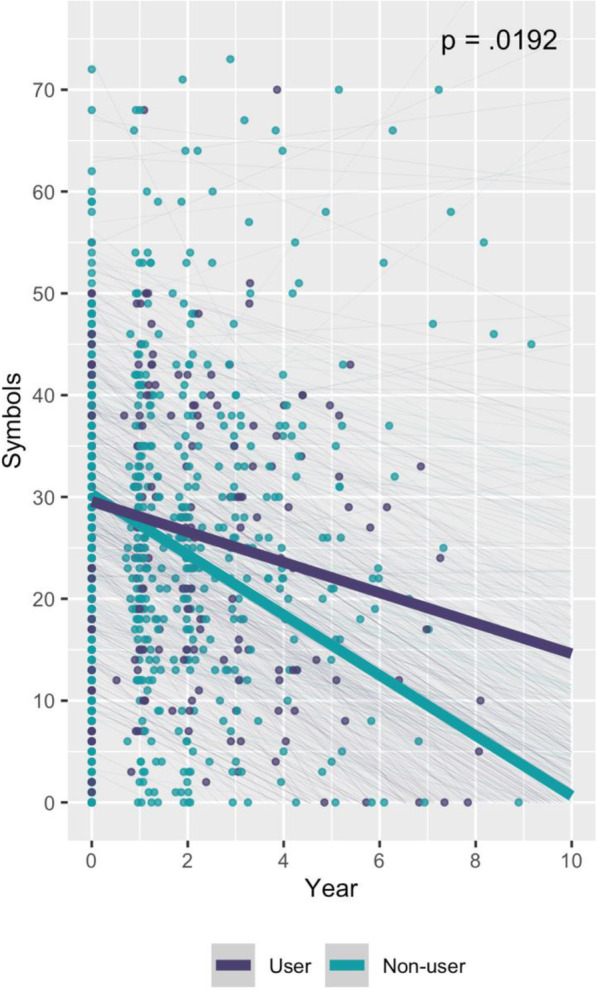
Association between LTRA use and psychomotor processing speed over time in the AD dementia group. Psychomotor processing speed was measured by the WAIS-R Digit Symbol Substitution Test. Thick lines represent the slope estimates for users and non-users over time, adjusted for covariates. Thin lines represent the adjusted slope estimates for each participant

**Fig. 2:**
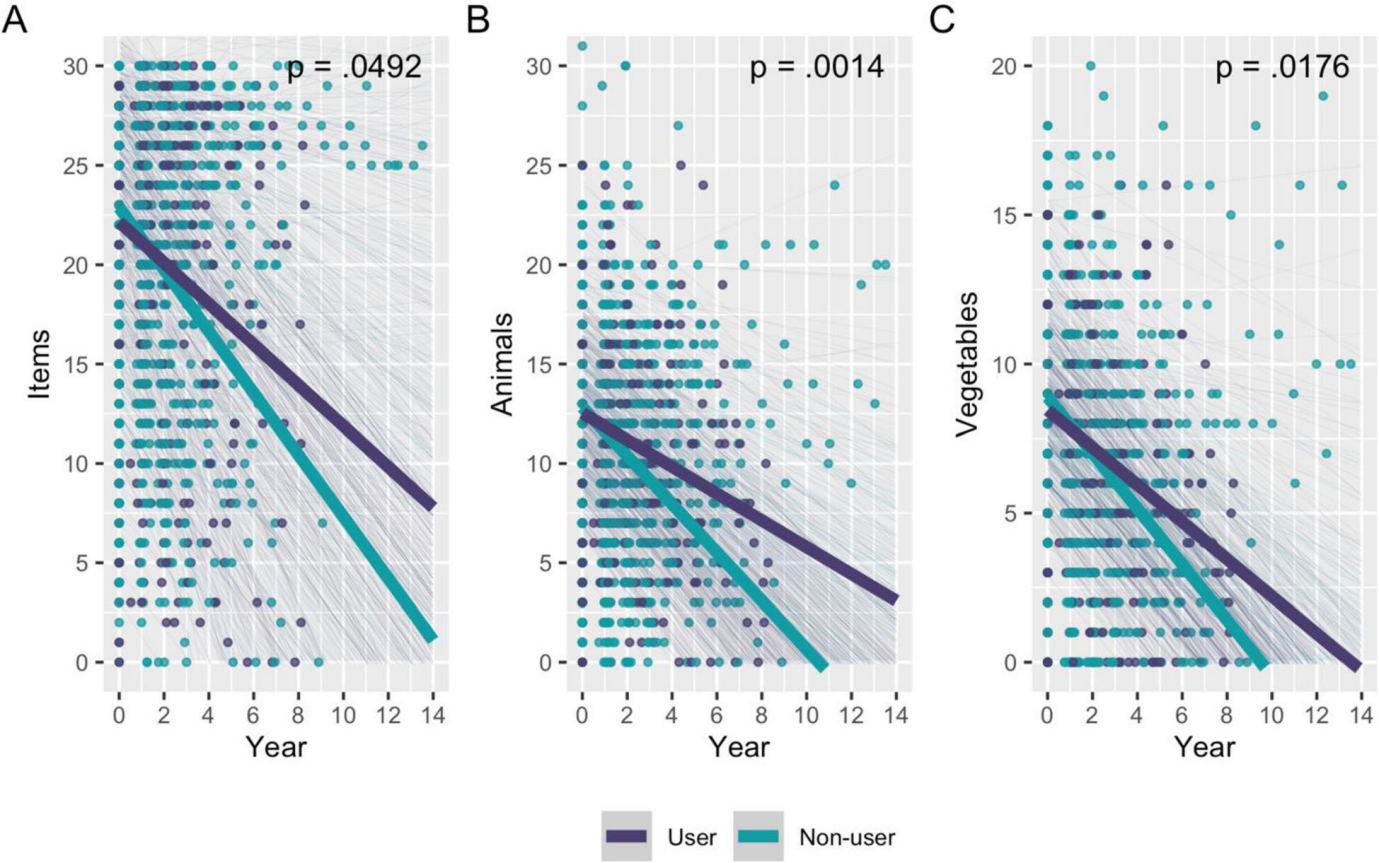
Association between LTRA use and language performance over time in the AD dementia group. Language performance was measured by **A** the Boston Naming Test, **B** animal naming, and **C** vegetable naming tests. Thick lines represent the slope estimates for users and non-users over time, adjusted for covariates. Thin lines represent the adjusted slope estimates for each participant

In the AD sample, LTRA use was associated with a slower decline in performance on tests of psychomotor processing speed and language (Table 4). Specifically, LTRA users showed better performance over time on the Digit Symbol Substitution Test (*Β* = 1.466 [0.253, 2.678] symbols/year; Fig. 1), Boston Naming Test (*B* = 0.529 [0.005, 1.053] items/year; Fig. 2A), animal naming (*Β* = 0.541 [0.215, 0.866] animals/year; Fig. 2B), and vegetable naming (*B* = 0.309 [0.056, 0.561] vegetables/year; Fig. 2C). These results remained significant after FDR correction. Effect sizes were relatively small (Table 4). LTRA use was not associated with a change in immediate (RR = 1.048 [0.972, 1.130]; Figure S2A) or delayed memory (RR = 1.065 [0.932, 1.217]; Figure S2B) performance.

**Table 4:** Associations between LTRA use and cognitive test performance over time in the AD dementia group

	***n***		RR or ***Β*** [95% CI]	***β*** [95% CI]	***z*** or ***t***	***p*** value
	User	Non-user				
**Logical memory**						
Immediate memory	141	419	RR = 1.048 [0.972, 1.130]	-	1.22	.2230
Delayed memory	140	416	RR = 1.065 [0.932, 1.217]	-	0.93	.3550
**Psychomotor processing speed**						
Digit Symbol Substitution Test	112	330	*B* = 1.466 [0.253, 2.678]	0.100 [0.017, 0.182]	2.37	.0192*
**Language**						
Boston Naming Test	141	420	*B* = 0.529 [0.005, 1.053]	0.069 [0.001, 0.137]	1.98	.0492*
Animal naming	143	423	*B* = 0.541 [0.215, 0.866]	0.101 [0.040, 0.162]	3.26	.0014*
Vegetable naming	143	422	*B* = 0.309 [0.056, 0.561]	0.079 [0.015, 0.143]	2.40	.0176*

*RR* rate ratio, *B* unstandardized coefficient, *z* for logical memory, and *t* for all other tests

^*^Significant at FDR 0.1

All results were consistent in a post hoc analysis excluding the zafirlukast users by matching users of montelukast to non-users of an LTRA (Tables S4, S5, and S6). In another post hoc analysis using CDR-SOB, the use of LTRAs was associated with a slower decline in clinical progression in the participants with AD dementia (*B* = −0.321 [−0.597, −0.046] points/year) and MCI (*B* = −0.200 [−0.380, −0.019] points/year) (Table S7).

In each analysis, a small number of participants who were included in the matching procedure did not provide cognitive data; however, inclusion of factors with SMD ≥ 0.1 as covariates in the outcome models for each comparison did not change the results.

## Discussion

In people with AD dementia, LTRA use was associated with better performance over time on tests for psychomotor processing speed and language but not memory. These longitudinal associations support the hypothesis that LTRAs may warrant investigation as a potential target for AD treatment. The lack of association with cognition among people with normal cognition and MCI does not provide evidence to support a role of LTRAs in slowing cognitive decline among people with normal cognition or MCI, although a small but significant effect on clinical progression was seen in both AD dementia and MCI groups.

The finding that LTRA use was associated with better performance in specific cognitive domains over time in AD dementia extends the results of a previous cross-sectional study, which found that the use of montelukast was associated with positive but non-significant differences in cognitive performance in people with undefined cognitive status [13]. That previous study suggested that the use of LTRAs may benefit cognition in older people, but it may have been underpowered due to sample sizes < 150 participants. The NACC data provided a larger sample of LTRA users than had been previously available, longitudinal assessments, data to characterize participants with respect to their cognitive status, and allowed us to carefully match users to non-users. By stratifying participants based on cognitive status, we show that associations between LTRA use and cognition over time were largest in people with pre-existing AD dementia. A positive association between LTRA use and performance on the Digit Symbol Substitution Test was also observed in people with MCI. Although the finding did not survive FDR correction, it is noteworthy that the effect size was smaller in MCI compared to AD, and absent in people with normal cognition. A previous cohort study found that the use of montelukast was associated with a lower risk of using a dementia medication and entering a nursing home [12], which may be consistent with our finding that LTRAs were associated with less cognitive decline and slower clinical progression in dementia, although the link would require further study. The results also suggest that the protective effects of LTRAs on cognition shown in some preclinical literature may be relevant in humans; for instance, montelukast improved cognition in a mouse model of AD [7] and in aged rats [6].

The use of LTRAs was not associated with better cognitive performance over time in people with normal cognition. In this group, the use of LTRAs was associated with a faster decline in immediate memory; however, the difference between users and non-users was < 1% and unlikely to be of clinical importance. Because users of an LTRA were matched for the use of other respiratory medications for allergy, COPD and asthma, they were using an additional medication, which may indicate a greater burden of allergic airway disease in the group of LTRA users. The approach was conservative and intended to avoid the overestimation of possible LTRA benefits; however, it is possible that the LTRA users could have had higher disease burden, which could also account for the small difference between groups seen in immediate memory performance.

Among people with AD dementia, the use of an LTRA was associated with better performance on tests of language and psychomotor processing speed. Although the use of LTRAs was positively associated with performance on tests for immediate and delayed memory in people with AD dementia, these associations were small and non-significant. The domains in which significant effects were found agree with the cross-sectional study described previously, which reported positive but non-significant associations with psychomotor processing speed and immediate free recall among older people using montelukast [13]. No studies thus far had examined language or delayed memory specifically. The results contrast a preclinical study, which showed that the administration of montelukast improved memory in aged rats [6]. A second study using the 5xFAD mouse model found that montelukast did not improve memory outcomes but improved learning outcomes [7], suggesting possible benefits of LTRAs on some cognitive domains. Although there may be interspecies differences, the present findings suggest that the preclinical findings may be relevant in humans and that the associations with LTRAs were larger for language and processing speed than for memory.

Given the different findings between AD dementia, cognitively normal or MCI groups, it might be speculated that the mechanisms underlying the benefits of LTRAs are more relevant later in the course of AD progression. In rodent models, administration of montelukast enhanced neurogenesis, increased blood-brain barrier (BBB) integrity, and reduced neuroinflammation [6, 7, 36], which may be of benefit in the context of accumulated AD pathology. Although montelukast may penetrate the BBB [37], it is unclear if it enters the brain at concentrations sufficient to have effects in the central nervous system in humans at the doses currently used in asthma treatment. At these concentrations, LTRAs may be acting primarily on the vasculature which may explain the beneficial effects observed in vascular-related cognitive domains such as psychomotor processing speed but not necessarily domains most specific to AD such as memory. Poor BBB penetration could also explain the relatively small effect sizes observed and effects seen only in AD where BBB disruption may be more appreciable. The observed effect sizes on cognition were small, which may indicate that the use of current LTRAs may have limited clinical utility; however, a post hoc analysis showed an effect on CDR-SOB in MCI and AD, suggesting that the use of LTRAs may have slowed dementia progression more broadly. Taken together, the results show that the leukotriene pathway warrants further investigation in AD treatment, either with currently available LTRAs (e.g., larger dose, increased frequency, or different route of administration) or with new investigational drugs that are targeting this pathway. Recently, a buccal film was produced which may help increase bioavailability of montelukast and achieve higher plasma concentrations [37]. Further studies are needed, particularly clinical trials, to determine potential efficacy and dose-response relationships between the LTRA use and cognition.

Cysteinyl leukotrienes are lipid mediators derived from arachidonic acid that activate CysLTR1 and GPR17 [9, 10]. They elicit a wide range of pro-inflammatory effects, including the expression of adhesion molecules and recruitment and activation of leukocytes [38, 39]. In addition to binding leukotriene receptors, LTRAs may have off-target effects; for instance, both montelukast and zafirlukast may inhibit soluble epoxide hydrolase (sEH) and modulate peroxisome proliferator activated receptor γ (PPARγ) at therapeutically relevant plasma concentrations [40]. These activities may be of benefit in AD; for instance, sEH inhibitors ameliorated AD pathology and improved behavior in the 5xFAD mouse model [41], and PPARγ agonists reduced cortical and hippocampal amyloid beta levels and improved spatial memory performance in the APP/PS1 mouse model [42]. Circulating metabolites of sEH were associated with cerebral small vessel disease and with poorer performance in associated cognitive domains [43, 44], offering one possible explanation for the preservation of psychomotor processing speed seen here. Further studies would be needed to elucidate the potential mechanisms of LTRAs in humans.

### Limitations

This study has important limitations. Although LTRA users were well matched to non-users on important demographics and use of other respiratory medications to minimize potential confounding by indication, clinician diagnoses related to the indication (e.g., asthma) were not available, and effects of unmeasured confounders (e.g., lung function) cannot be ruled out. Since patients were matched for other respiratory medications, LTRA users were using an LTRA in addition to those medications, and they may have had more severe respiratory disease overall; however, these differences may have biased the results towards the null, suggesting that the effect sizes may have been conservative in their estimates. Although LTRAs included montelukast and zafirlukast, only seven participants (0.2%) used zafirlukast. A sensitivity analysis which included only montelukast users did not change the results, but more data would be required to generalize the conclusions to zafirlukast users. Our analyses could not account for adherence, frequency of usage, duration of usage, or dosage of medications since these data were not collected in the UDS. Higher doses of montelukast resulted in better learning performance in 5xFAD mice, suggesting that a dose-response relationship may exist [7]. Future studies should examine possible dose effects and compare short- vs. long-term LTRA use. Although the use of prescribed allergy medications (decongestants, antihistamines, and nasal corticosteroids) was matched for, the use of over-the-counter medications was not collected systematically, and therefore, some exposures to allergy medications may not have been accounted for. Drug exposures before entering NACC were not recorded, and observations where medication data was not provided were excluded from the analyses, which could have contributed to misclassification of the exposure group. Although MRI data were available in NACC, they were not available in a sufficient number of participants to explore the associations between the LTRA use and imaging measures. AD was identified using clinical criteria due to insufficient biomarker or post-mortem data in the NACC to confirm diagnoses. Future studies might examine biomarker confirmed subgroups and also the effects of LTRA use on biomarker progression and imaging measures. Participants were recruited by many ADRC sites, but they may not be representative of the general population in certain factors such as educational attainment, socioeconomic status, and proportions of Whites and women, which could reduce generalizability.

## Conclusion

The use of LTRAs was associated with slower decline in specific domains of cognitive performance and slower clinical decline in AD dementia. These results provide clinical evidence suggesting that the leukotriene pathway may be relevant in the progression of AD and that there may be potential benefits of treatments that target the pathway.

## Supplementary Information

**Additional file 1.** Participant selection flow chart, figure showing logical memory performance over time in the AD dementia group, table of respiratory medications, table of participant characteristics before propensity score matching, and tables for post-hoc analyses.

## Data Availability

The data analyzed in the current study are available through the NACC, https://naccdata.org/.

## Abbreviations

AD: Alzheimer’s disease
ADRC: Alzheimer’s Disease Research Centers
*APOE*: Apolipoprotein E
BBB: Blood-brain barrier
COPD: Chronic obstructive pulmonary disease
CysLTR1: Cysteinyl leukotriene receptor 1
FDR: False discovery rate
GPR17: G-coupled protein receptor 17
LTRA: Leukotriene receptor antagonist
MCI: Mild cognitive impairment
NACC: National Alzheimer’s Coordinating Center
PPARγ: Peroxisome proliferator activated receptor γ
RR: Rate ratio
sEH: Soluble epoxide hydrolase
SMD: Standardized mean difference
UDS: Uniform Data Set
