# Supplementary material for: Leukotriene receptor antagonist use and cognitive decline in normal cognition, mild cognitive impairment, and Alzheimer’s dementia
Source: Alzheimers Res Ther. 2021 Sep 3;13:147. doi: 10.1186/s13195-021-00892-7 (PMC8418104; doi:10.1186/s13195-021-00892-7)
Supplement: Supplementary file 1 — Additional file 1. Participant selection flow chart, figure showing logical memory performance over time in the AD dementia group, table of respiratory medications, table of participant characteristics before propensity score matching, and tables for post-hoc analyses. [file 13195_2021_892_MOESM1_ESM.docx]

**Supplementary Materials**


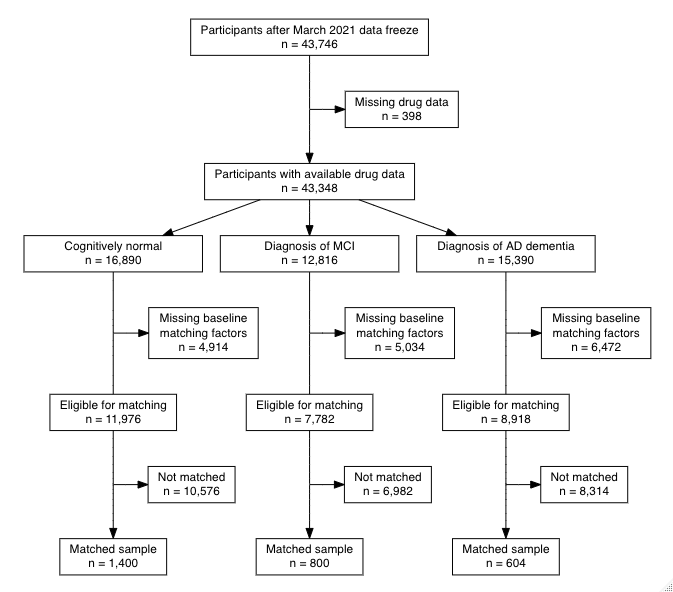


Figure S1. Flowchart of participant selection.


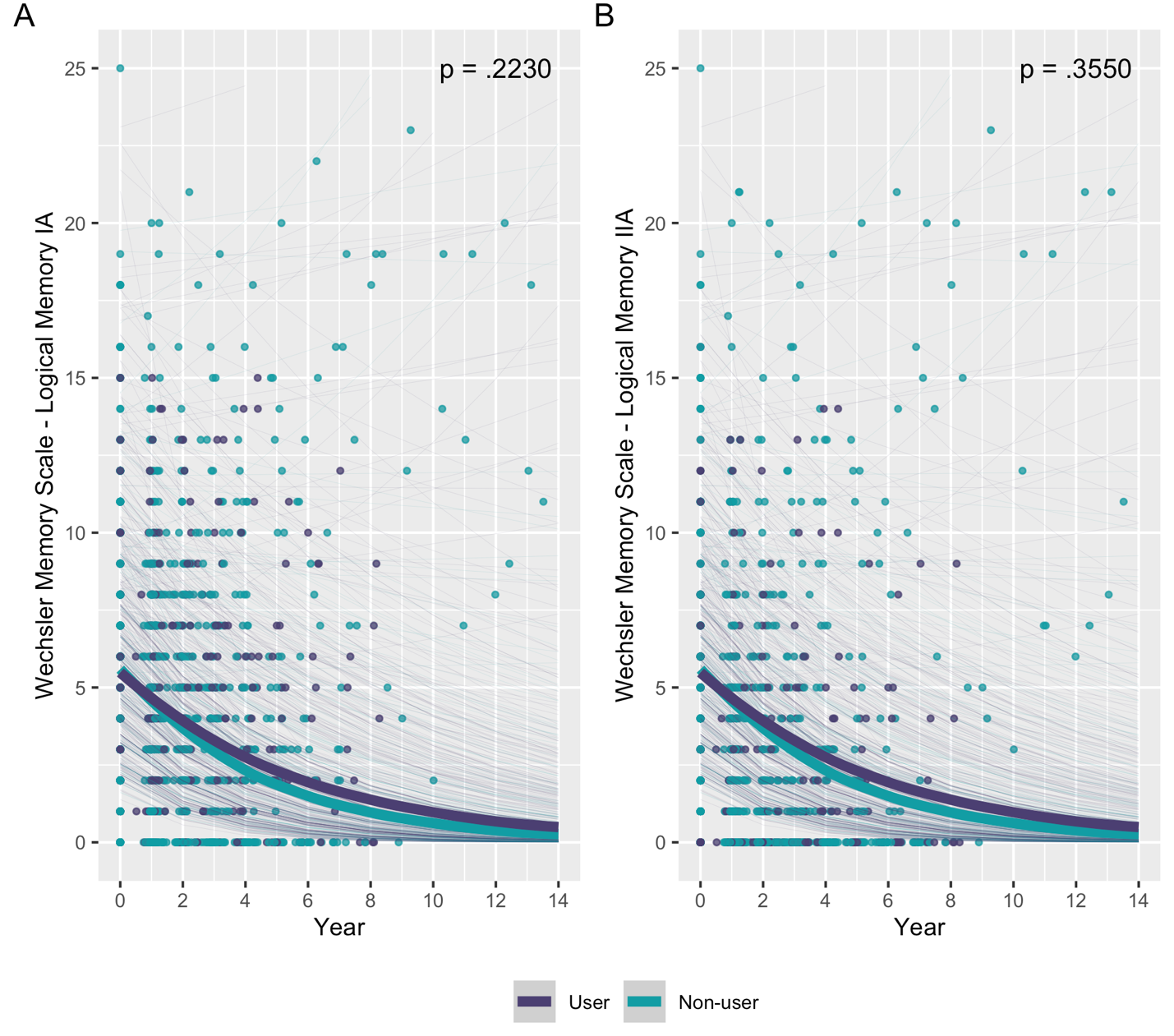


Figure S2. Association between LTRA use and logical memory performance over time in the AD dementia group. Logical memory performance was measured by the Wechsler Memory Scale – Logical Memory test (A) immediate memory IA and (B) delayed memory IIA. Thick lines represent the slope estimates for users and non-users over time, adjusted for covariates. Thin lines represent the adjusted slope estimates for each participant.

Table S1. Respiratory medications included in matching factors.

| **Medication type** | **Drug class** | **Drug name** |
| --- | --- | --- |
| Rescue inhaler for asthma | Short-acting β-agonists (SABAs) | - Albuterol - Levalbuterol |
| Inhalers for asthma maintenance | Combination inhalers | - Budesonide-formoterol - Fluticasone-vilanterol - Fluticasone-salmeterol - Formoterol-mometasone |
|  | Corticosteroids | - Beclomethasone - Budesonide - Ciclesonide - Fluticasone - Mometasone - Flunisolide - Triamcinolone |
|  | Long-acting β-agonists (LABAs) | - Formoterol - Salmeterol - Arformoterol |
| COPD-specific medications | Combination inhalers | - Albuterol-ipratropium - Ormoterol-glycopyrrolate - Olodaterol-tiotropium - Umeclidinium-vilanterol - Aclidinium-formoterol |
|  | Short-acting muscarinic antagonists (SAMAs) | - Ipratropium |
|  | Long- acting muscarinic antagonists (LAMAs) | - Aclidinium - Umeclidinium - Tiotropium - Glycopyrronium - Umeclidinium |
|  | Long-acting β-agonists (LABAs) | - Olodaterol - Indacaterol |
| Allergies | Decongestants | - Oxymetazoline - Phenylephrine - Pseudoephedrine |
|  | Antihistamines | - Diphenhydramine - Chlorpheniramine - Cetirizine - Desloratadine - Fexofenadine - Levocetirizine - Loratadine - Azelastine nasal - Olopatadine nasal |
|  | Corticosteroids | - Beclomethasone nasal - Budesonide nasal - Ciclesonide nasal - Fluticasone nasal - Mometasone nasal - Flunisolide nasal - Triamcinolone nasal |

Table S2. Baseline participant characteristics by LTRA use in normal, MCI, and AD participants before propensity score matching.

|  | **Normal** | | | **MCI** | | | **AD** | | |
| --- | --- | --- | --- | --- | --- | --- | --- | --- | --- |
|  | **User (n = 350)** | **Non-user  (n = 11626)** | **SMD** | **User (n = 200)** | **Non-user  (n = 7582)** | **SMD** | **User (n = 151)** | **Non-user  (n = 8767)** | **SMD** |
| **Baseline demographics** | | | | | | | | | |
| Age (years) | 70.30 (9.71) | 70.67 (10.62) | 0.036 | 73.75 (8.86) | 74.36 (9.53) | 0.066 | 75.35 (9.73) | 75.00 (9.93) | 0.036 |
| Female | 261 (74.6%) | 7550 (64.9%) | 0.211 | 110 (55.0%) | 3813 (50.3%) | 0.094 | 93 (61.6%) | 4668 (53.2%) | 0.169 |
| BMI (kg/m^2^) | 29.41 (6.21) | 27.32 (5.35) | 0.361 | 27.82 (5.54) | 26.92 (5.03) | 0.170 | 27.03 (6.05) | 26.14 (4.77) | 0.163 |
| Education (years) | 15.77 (3.29) | 15.85 (2.95) | 0.028 | 15.23 (3.32) | 15.30 (3.38) | 0.020 | 13.87 (4.51) | 14.56 (3.62) | 0.169 |
| Smoking history | 158 (45.1%) | 5075 (43.7%) | 0.030 | 86 (43.0%) | 3415 (45.0%) | 0.041 | 60 (39.7%) | 3735 (42.6%) | 0.058 |
| **Dementia measures** | | | | | | | | | |
| CDR global score |  |  | 0.040 |  |  | 0.023 |  |  | 0.206 |
| 0 | 316 (90.3%) | 10595 (91.1%) |  | 25 (12.5%) | 909 (12.0%) |  | 0 (0.0%) | 37 (0.4%) |  |
| 0.5 | 34 (9.7%) | 1027 (8.8) |  | 172 (86.0%) | 6542 (86.3%) |  | 58 (38.4%) | 3399 (38.8%) |  |
| 1 | 0 (0.0%) | 0 (0.0%) |  | 3 (1.5%) | 131 (1.7%) |  | 61 (40.4%) | 3864 (44.1%) |  |
| 2 | 0 (0.0%) | 0 (0.0%) |  | 0 (0.0%) | 0 (0.0%) |  | 28 (18.5%) | 1099 (12.5%) |  |
| 3 | 0 (0.0%) | 0 (0.0%) |  | 0 (0.0%) | 0 (0.0%) |  | 4 (2.6%) | 368 (4.2%) |  |
| *APOE* ε4 carrier | 116 (33.1%) | 3587 (30.9%) | 0.049 | 84 (42.0%) | 3150 (41.5%) | 0.009 | 81 (53.6%) | 5024 (57.3%) | 0.074 |
| Vascular dementia | 0 (0.0%) | 0 (0.0%) | <0.001 | 7 (3.5%) | 184 (2.4%) | 0.063 | 6 (4.0%) | 221 (2.5%) | 0.082 |
| AD medication use | 0 (0.0%) | 0 (0.0%) | <0.001 | 52 (26.0%) | 1464 (19.3%) | 0.160 | 100 (66.2%) | 5431 (61.9%) | 0.089 |
| **Other respiratory medication use** | | | | | | | | | |
| Allergy medications | 152 (43.4%) | 1137 (9.8%) | 0.824 | 82 (41.0%) | 656 (8.7%) | 0.808 | 51 (33.8%) | 587 (6.7%) | 0.716 |
| COPD medications | 27 (7.7%) | 111 (1.0%) | 0.337 | 27 (13.5%) | 97 (1.3%) | 0.480 | 17 (11.3%) | 121 (1.4%) | 0.415 |
| Rescue inhaler for asthma | 82 (23.4%) | 334 (2.9%) | 0.638 | 40 (20.0%) | 209 (2.8%) | 0.564 | 36 (23.8%) | 169 (1.9%) | 0.692 |
| Maintenance inhaler for asthma | 136 (38.9%) | 365 (3.1%) | 0.976 | 78 (39.0%) | 296 (3.9%) | 0.946 | 49 (32.5%) | 219 (2.5%) | 0.858 |

Continuous variables expressed as mean (standardized deviation); categorical variables expressed as counts (proportion).

Table S3. Baseline participant characteristics by montelukast use in normal, MCI, and AD groups following propensity score matching in post-hoc analysis.

|  | **Normal** | | | **MCI** | | | **AD** | | |
| --- | --- | --- | --- | --- | --- | --- | --- | --- | --- |
|  | **User (n = 346)** | **Non-user  (n = 1038)** | **SMD** | **User (n = 198)** | **Non-user  (n = 594)** | **SMD** | **User (n = 150)** | **Non-user  (n = 450)** | **SMD** |
| **Baseline demographics** | | | | | | | | | |
| Age (years) | 70.29 (9.74) | 70.38 (9.59) | 0.009 | 73.80 (8.86) | 73.90 (8.74) | 0.011 | 75.35 (9.76) | 75.28 (10.10) | 0.007 |
| Female | 258 (74.6%) | 784 (75.5%) | 0.022 | 109 (55.1%) | 317 (53.4%) | 0.034 | 92 (61.3%) | 263 (58.4%) | 0.059 |
| BMI (kg/m^2^) | 29.44 (6.23) | 29.72 (6.78) | 0.043 | 27.81 (5.57) | 27.96 (6.18) | 0.026 | 27.05 (6.07) | 26.94 (5.09) | 0.019 |
| Education (years) | 15.75 (3.29) | 15.79 (2.83) | 0.016 | 15.24 (3.33) | 15.19 (3.25) | 0.016 | 13.88 (4.52) | 14.03 (3.78) | 0.035 |
| Smoking history | 155 (44.8%) | 469 (45.2%) | 0.008 | 85 (42.9%) | 270 (45.5%) | 0.051 | 60 (40.0%) | 197 (43.8%) | 0.077 |
| **Dementia measures** | | | | | | | | | |
| CDR global score |  |  | 0.026 |  |  | 0.065 |  |  | 0.120 |
| 0 | 314 (90.8%) | 934 (90.0%) |  | 25 (12.6%) | 72 (12.1%) |  | 0 (0.0%) | 0 (0.0%) |  |
| 0.5 | 32 (9.2%) | 104 (10.0%) |  | 170 (85.9%) | 517 (87.0%) |  | 57 (38.0%) | 197 (43.8%) |  |
| 1 | 0 (0.0%) | 0 (0.0%) |  | 3 (1.5%) | 5 (0.8%) |  | 61 (40.7%) | 168 (37.3%) |  |
| 2 | 0 (0.0%) | 0 (0.0%) |  | 0 (0.0%) | 0 (0.0%) |  | 28 (18.7%) | 73 (16.2%) |  |
| 3 | 0 (0.0%) | 0 (0.0%) |  | 0 (0.0%) | 0 (0.0%) |  | 4 (2.7%) | 12 (2.7%) |  |
| *APOE* ε4 carrier | 116 (33.5%) | 341 (32.9%) | 0.014 | 83 (41.9%) | 244 (41.1%) | 0.017 | 81 (54.0%) | 251 (55.8%) | 0.036 |
| Vascular dementia | 0 (0.0%) | 0 (0.0%) | <0.001 | 7 (3.5%) | 28 (4.7%) | 0.059 | 6 (4.0%) | 14 (3.1%) | 0.048 |
| AD medication use | 0 (0.0%) | 0 (0.0%) | <0.001 | 51 (25.8%) | 152 (25.6%) | 0.004 | 100 (66.7%) | 300 (66.7%) | <0.001 |
| **Other respiratory medication use** | | | | | | | | | |
| Allergy medications | 148 (42.8%) | 467 (45.0%) | 0.045 | 82 (41.4%) | 240 (40.4%) | 0.021 | 50 (33.3%) | 160 (35.6%) | 0.047 |
| COPD medications | 27 (7.8%) | 57 (5.5%) | 0.093 | 27 (13.6%) | 65 (10.9%) | 0.082 | 17 (11.3%) | 47 (10.4% | 0.029 |
| Rescue inhaler for asthma | 81 (23.4%) | 177 (17.1%) | 0.159 | 40 (20.2%) | 129 (21.7%) | 0.037 | 36 (24.0%) | 92 (20.4%) | 0.086 |
| Maintenance inhaler for asthma | 136 (39.3%) | 349 (33.6%) | 0.118 | 78 (39.4%) | 211 (35.5%) | 0.080 | 48 (32.0%) | 137 (30.4%) | 0.034 |

Continuous variables expressed as mean (standardized deviation); categorical variables expressed as counts (proportion).

Table S4. Associations between montelukast use and cognitive test performance over time in the normal cognition group in post-hoc analysis excluding the zafirlukast users.

|  | **n** | | **RR or Β^a^ [95% CI]** | **β [95% CI]** | ***z* or *t*^b^** | ***p-*value** |
| --- | --- | --- | --- | --- | --- | --- |
|  | **User** | **Non-user** |  |  |  |  |
| **Logical memory** | | | | | | |
| Immediate memory | 341 | 1028 | RR = 0.992 [0.985, 0.998] | - | -2.57 | .0101* |
| Delayed memory | 341 | 1028 | RR = 0.995 [0.987, 1.003] | - | -1.41 | .1903 |
| **Psychomotor processing speed** | | | | | | |
| Digit Symbol Substitution Test | 275 | 733 | B = 0.155 [-0.102, 0.413] | 0.012 [-0.008, 0.031] | 1.18 | .2384 |
| **Language** | | | | | | |
| Boston Naming Test | 340 | 1029 | B = -0.019 [-0.081, 0.042] | -0.006 [-0.026, 0.014] | -0.62 | .5374 |
| Animal naming | 341 | 1031 | B = 0.042 [-0.060, 0.144] | 0.007 [-0.011, 0.025] | 0.80 | .4231 |
| Vegetable naming | 341 | 1027 | B = -0.019 [-0.096, 0.059] | -0.004 [-0.022, 0.013] | -0.47 | .6386 |

^a^RR = rate ratio; and B = unstandardized coefficient.

^b^*z* for logical memory and *t* for all other tests.

*Significant at FDR 0.1.

Table S5. Associations between montelukast use and cognitive test performance over time in the MCI group in post-hoc analysis excluding the zafirlukast users.

|  | **n** | | **RR or Β^a^ [95% CI]** | **β [95% CI]** | ***z* or *t*^b^** | ***p-*value** |
| --- | --- | --- | --- | --- | --- | --- |
|  | **User** | **Non-user** |  |  |  |  |
| **Logical memory** | | | | | | |
| Immediate memory | 195 | 576 | RR = 1.015 [0.991, 1.039] | - | 1.20 | .2312 |
| Delayed memory | 195 | 575 | RR = 1.030 [0.990, 1.072] | - | 1.46 | .1443 |
| **Psychomotor processing speed** | | | | | | |
| Digit Symbol Substitution Test | 148 | 427 | B = 0.711 [0.045, 1.377] | 0.0568 [0.004, 0.111] | 2.09 | .0375 |
| **Language** | | | | | | |
| Boston Naming Test | 195 | 578 | B = 0.187 [-0.054, 0.428] | 0.035 [-0.010, 0.080] | 1.52 | .1293 |
| Animal naming | 195 | 578 | B = 0.184 [-0.051, 0.419] | 0.033 [-0.009, 0.074] | 1.54 | .1254 |
| Vegetable naming | 195 | 575 | B = 0.033 [-0.133, 0.199] | 0.008 [-0.031, 0.046] | 0.39 | .7008 |

^a^RR = rate ratio; and B = unstandardized coefficient.

^b^*z* for logical memory and *t* for all other tests.

*Significant at FDR 0.1.

Table S6. Associations between montelukast use and cognitive test performance over time in AD group in post-hoc analysis excluding the zafirlukast users.

|  | **n** | | **RR or Β^a^ [95% CI]** | **β [95% CI]** | ***z* or *t*^b^** | ***p-*value** |
| --- | --- | --- | --- | --- | --- | --- |
|  | **User** | **Non-user** |  |  |  |  |
| **Logical memory** | | | | | | |
| Immediate memory | 140 | 416 | RR = 1.049 [0.972, 1.131] | - | 1.24 | .2160 |
| Delayed memory | 139 | 413 | RR = 1.067 [0.933, 1.221] | - | 0.95 | .3410 |
| **Psychomotor processing speed** | | | | | | |
| Digit Symbol Substitution Test | 112 | 327 | B = 1.477 [0.260, 2.694] | 0.100 [0.018, 0.182] | 2.38 | .0188* |
| **Language** | | | | | | |
| Boston Naming Test | 140 | 417 | B = 0.541 [0.016, 1.065] | 0.071 [0.003, 0.139] | 2.02 | .0444* |
| Animal naming | 142 | 420 | B = 0.554 [0.218, 0.870] | 0.101 [0.041, 0.162] | 3.27 | .0014* |
| Vegetable naming | 142 | 419 | B = 0.315 [0.062, 0.568] | 0.081 [0.016, 0.145] | 2.44 | .0159* |

^a^RR = rate ratio; and B = unstandardized coefficient.

^b^*z* for logical memory and *t* for all other tests.

*Significant at FDR 0.1.

Table S7. Association between montelukast use and CDR-SB over time in post-hoc analysis.

| **Cognitive group** | **n** | | **Β [95% CI]** | **β [95% CI]** | ***t*** | ***p* value** |
| --- | --- | --- | --- | --- | --- | --- |
|  | **User** | **Non-user** |  |  |  |  |
| MCI | 200 | 600 | -0.200 [-0.380, -0.019] | -0.065 [-0.125, -0.006] | -2.17 | 0.0305 |
| AD | 151 | 453 | -0.321 [-0.597, -0.046] | -0.065 [-0.120, -0.009] | -2.28 | 0.0234 |
